# Multispectral photoacoustic microscopy and optical coherence tomography using a single supercontinuum source

**DOI:** 10.1016/j.pacs.2017.11.002

**Published:** 2017-11-26

**Authors:** M. Bondu, M.J. Marques, P.M. Moselund, G. Lall, A. Bradu, A. Podoleanu

**Affiliations:** a University of Kent, Applied Optics Group, School of Physical Sciences, Ingram building, Canterbury CT2 7NH, United Kingdom; b NKT Photonics A/S, Blokken 84, Birkeroed 3460, Denmark; c Medway School of Pharmacy, University of Kent, Chatham ME4 4TB, United Kingdom

**Keywords:** Multimodal imaging, Multispectral imaging, Photoacoustic, Optical coherence tomography, Supercontinuum

## Abstract

We report on the use of a single supercontinuum (SC) source for multimodal imaging. The 2-octave bandwidth (475–2300 nm) makes the SC source suitable for optical coherence tomography (OCT) as well as for multispectral photoacoustic microscopy (MPAM). The IR band centered at 1310 nm is chosen for OCT to penetrate deeper into tissue with 8 mW average power on the sample. The 500–840 nm band is used for MPAM. The source has the ability to select the central wavelength as well as the spectral bandwidth. An energy of more than 35 nJ within a less than 50 nm bandwidth is achieved on the sample for wavelengths longer than 500 nm. In the present paper, we demonstrate the capabilities of such a multimodality imaging instrument based on a single optical source. *In vitro* mouse ear B-scan images are presented.

## 1 Introduction

Optical coherence tomography (OCT) and photoacoustic microscopy (PAM) provide complementary contrasts, which can be combined to benefit biomedical research and preclinical studies. OCT allows structure reconstruction via backscattered signal due to the scattering properties of the sample being investigated. PAM is based on tissue absorption properties by optical excitation and ultrasonic detection. Both techniques offer real-time and non-invasive imaging. Combined OCT-PAM systems have already been reported for a wide range of applications such as dermal or retinal imaging [1], [2], [3], [4], [5].

In most of the previous work carried out on bimodal OCT-PAM, two different light sources are used to accommodate the requirements of each modality. OCT employs broadband light sources, the most common of these being super-luminescent diodes (SLD). Recent developments of low noise supercontinuum sources (NKT Photonics A/S) made such sources attractive to ultra-high resolution OCT [6] as well as visible OCT [7], [8] since they offer a spectral range from 475 nm to 2300 nm. No other light source can cover such a wide bandwidth. PAM typically requires a light source with a pulse duration of a few nanoseconds because of thermal and stress confinement of the excitation beam in the sample. PAM commonly uses single-wavelength lasers as the excitation source, such as Nd:YAG lasers at 1064 nm [9] or its frequency-doubled counterpart at 532 nm [10], or dye lasers [11]. In order to perform multispectral PAM (MPAM), one can use multiple lasers [12], optical parametric oscillators [13] (at the cost of price and speed), stimulated Raman scattering fiber lasers [14], [15] (discrete wavelengths with limited choice) or supercontinuum sources [5], [16], [17] (wide wavelength range with limited energy). Previously reported supercontinuum sources for MPAM delivered too little energy per pulse to image biological samples [16], [17], more than 50 nJ within a bandwidth of less than 50 nm is typically required. Preliminary work targeting the enhancement in the pulse energy of supercontinuum sources (>100 nJ) has been reported [18], [19].

Here a commercial supercontinuum source from NKT Photonics A/S (SuperK Compact) that offers more than 35 nJ per bandwidth of less than 50 nm over a broad wavelength range extending from 500 nm to 800 nm is used. The source is cost effective, compact and reliable. In addition, such a source can be used for OCT and can offer high resolution due to the large optical bandwidth available. To our knowledge, this is the first report on a combined OCT-MPAM system that offers fast volumetric imaging as well as spectroscopic measurements within a wide range of wavelengths covering the visible and extending from 500 nm to 840 nm with comparable energy to those of single wavelength lasers. This paper is an extension and continuation of the preliminary OCT images and spectroscopic photoacoustic measurements presented in our previous work [20]. Visible light is used in this paper because we intend to target hemoglobin, which absorbs mainly between 500 nm and 600 nm. This is the most imaged absorber for PAM, reported for MPAM alone [17] or combined with the structural information of OCT [1], [2], [3], [4], [5].

After some primary work to illustrate the system capabilities, we present in Section 3.3 *in vitro* images of a mouse ear. The determination of hemoglobin oxygen saturation is of great interest, and due to the multispectral capabilities of the system, we are able to estimate the oxygen saturation of the blood present in the mouse ear.

## 2 Material and methods

**Fig. 1:**
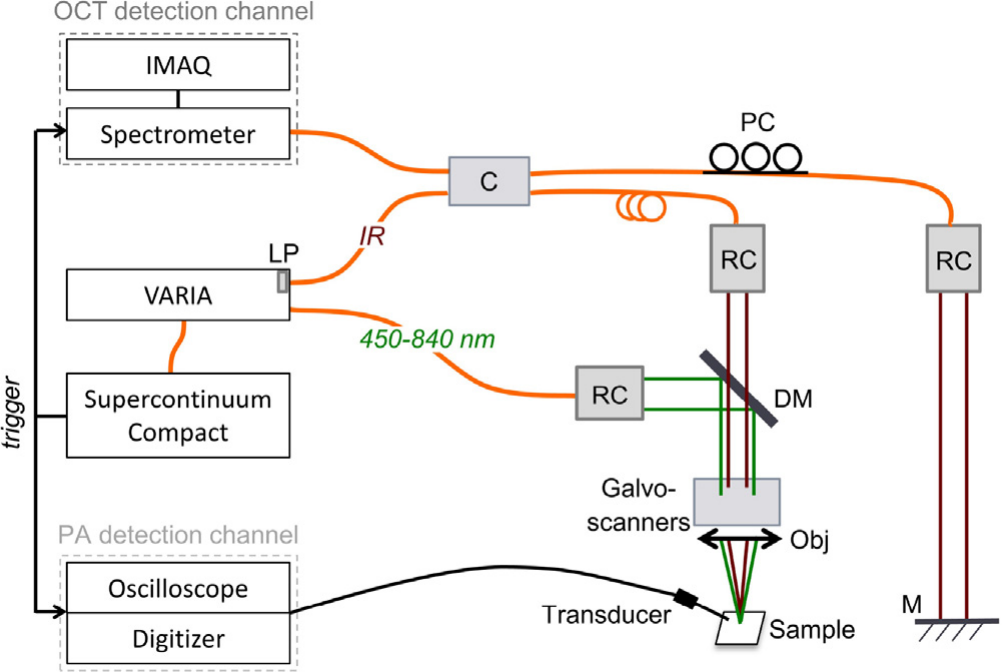
Schematic of the combined OCT-MPAM system. C: 50/50 coupler, DM: dichroic mirror, LP: longpass filter, M: mirror (OCT reference arm), Obj: objective lens, PC: polarization controller, RC: reflective collimators.

**Fig. 2:**
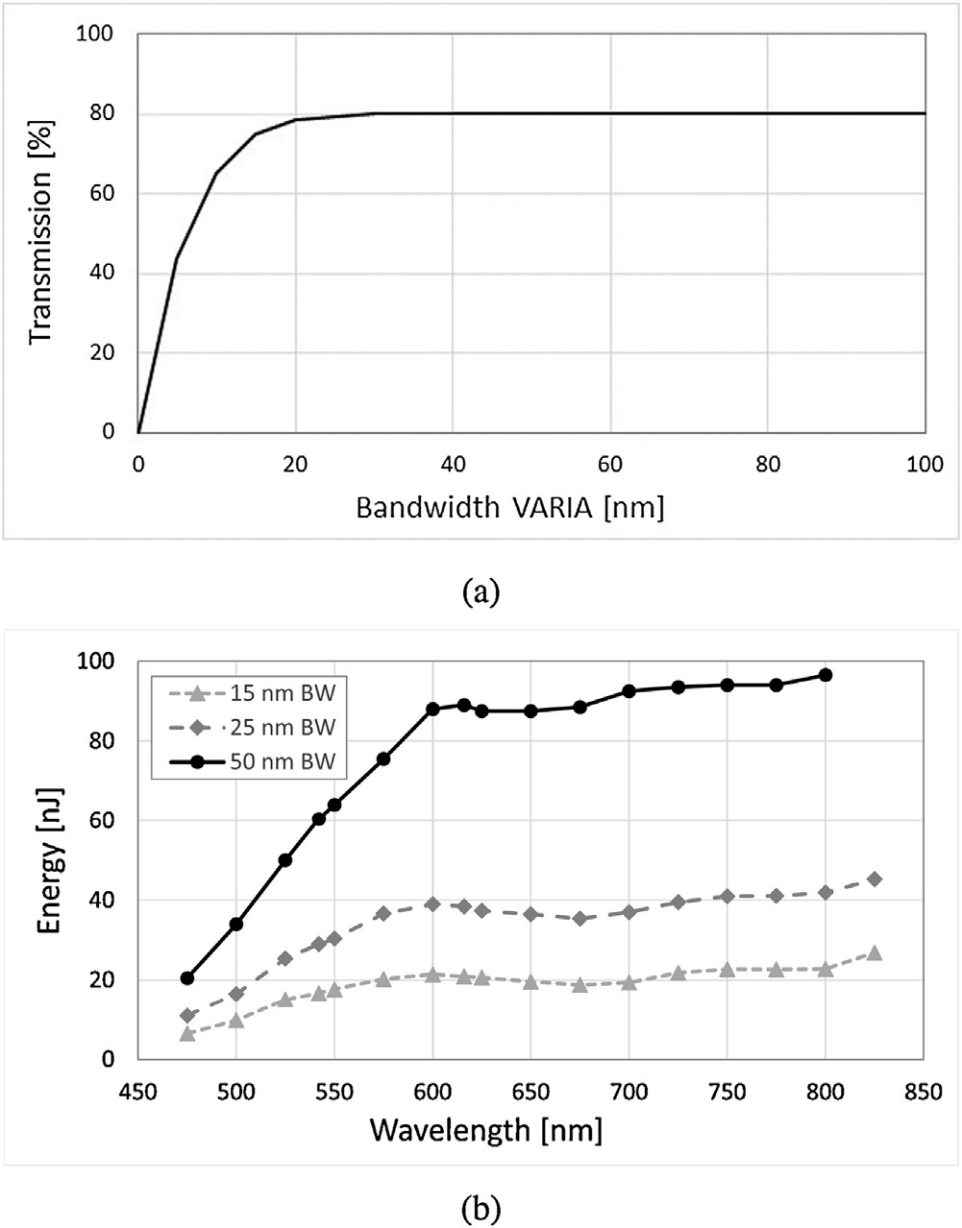
Visible channel of the system with: (a) the transmission through the VARIA depending on the bandwidth and (b) the energy per pulse measured on the sample for different central wavelengths and bandwidths.

**Fig. 3:**
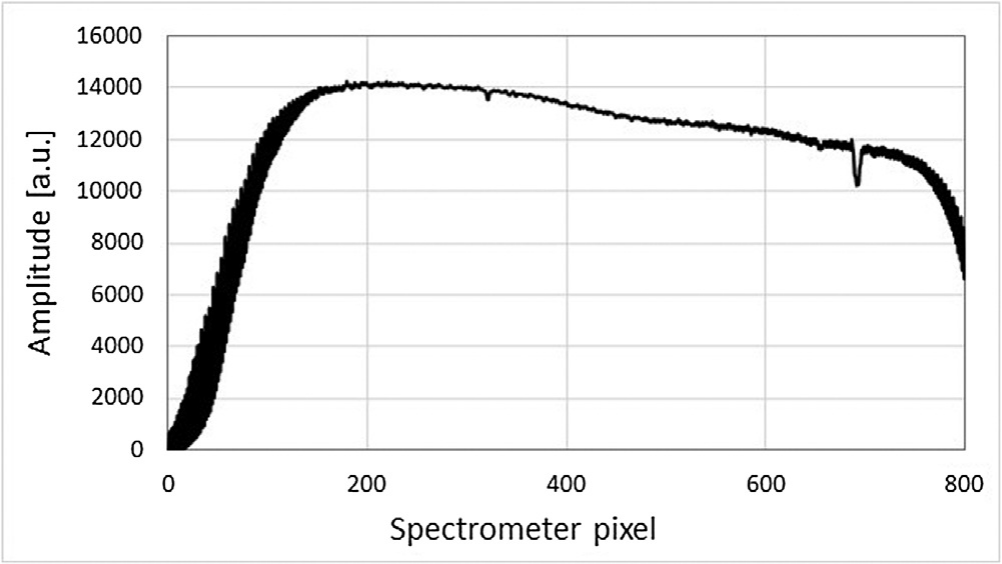
Spectrum recorded by the in-house built spectrometer corresponding to the reference arm of the system only.

Fig. 1 represents the schematic of the experimental set-up. The supercontinuum source (Compact, NKT Photonics A/S) is used in combination with a dual band filter (VARIA, NKT Photonics A/S) which splits the spectrum into a short wavelength band and an IR band. The short wavelength band, covering a broad range from 450 nm to 840 nm, is used for MPAM. The filter selects a central wavelength and a bandwidth within this range, which can be tuned over time (the switching time between two spectral ranges is less than 5 s). Fig. 2(a) shows the transmission of the filter depending on the bandwidth and Fig. 2(b) the energy on sample for different central wavelengths and bandwidths. The IR band covers an ultra-broad range extending 850–2300 nm. The short wavelength band and the IR band are fiber-delivered to facilitate the implementation of the combined system (Connect FD7 non-PM and FD5 non-PM respectively, NKT Photonics A/S). A 50/50 fiber coupler is used for the interferometer (TW1300R5A2, Thorlabs) with a polarization controller in the reference arm only. The FD5 fiber and the coupler are working as bandpass filters. However due to the presence of supercontinuum pump power at 1060 nm, further filtering is required, which is achieved by using a longpass optical filter with a cut-off wavelength of 1150 nm (FEL1150, Thorlabs) positioned between the VARIA box and the Connect FD5 (using a custom accessory). Thus, a spectrum centered at 1310 nm for the OCT channel with a power of 8 mW on the sample is achieved. Silver-coated reflective fiber collimators are used to ensure achromaticity. In the sample arm, the two beams, used for OCT and PAM, are both conveyed via a dichroic mirror (DMLP950, Thorlabs), then reflected by two galvo-scanners that allow raster scanning of the sample in the lateral directions (6220H, Cambridge Technology). The scanners limit the beam diameters to a maximum of 5 mm, therefore ∼4 mm beams are used in the sample arm, determined by the numerical aperture of the optical fibers and the focal length of the collimators (RC04APC-01, Thorlabs). A different collimator (RC08APC-01, Thorlabs) has been used in the reference arm of the OCT channel because of availability. However the same collimator could have been used in both arms. An uncoated lens (LA1027, Thorlabs) focuses the two beams on the sample. A 3D translation stage supports the sample horizontally and facilitates its alignment. In terms of detection, the OCT bandwidth is limited to 90 nm by our in-house built spectrometer, which consists of a diffraction transmission grating (HP 1145 l/mm @ 1310 nm, Wasatch Photonics) and a line scan camera (SU1014-LDHI.7RT-0500/L, Goodrich). Fig. 3 shows the spectrometer readout when signal from the reference arm is applied only. An IMAQ board (PCI-1428, National Instruments) is used for the OCT detection channel. The photoacoustic signal is detected through water by a customized unfocused needle ultrasonic transducer (40.3 MHz center frequency, 90% bandwidth at −6 dB, 0.4 mm diameter active element, University of Southern California). The electric signal hence generated is then amplified (ZFL-500LN+, Mini Circuits) and either digitized at a sampling rate of 200 MHz (PCI-5124, National Instruments) or read by the oscilloscope for the measurements presented in Section 3.2.

Assuming a Gaussian-shaped source spectrum, the 90 nm bandwidth of the spectrometer determines a theoretical axial resolution in air of 8.4 μm, in the OCT channel. However, experimentally, an axial resolution of 12.3 μm was measured with no apodization of the channeled spectra, and 17 μm when applying a Hamming window (usually employed for filtering fast transitions). The axial resolution of PAM is determined by the bandwidth of the transducer: 36 MHz, which gives a theoretical axial resolution of 36 μm. We obtained an experimental axial resolution of approximately 34 μm by measuring the width of the A-scan peak corresponding to the chrome layer of an USAF target (<1 μm). By imaging the USAF target, we measured the experimental lateral resolution of both modalities. The lateral resolution is slightly dependent on the wavelength; a theoretical lateral resolution of 4–7.5 μm is calculated for wavelengths covering the 450–840 nm wavelength range, and 8.5 μm for the OCT at 1310 nm. We could resolve element 4 from group 6 with OCT, corresponding to a lateral resolution of 10.1 μm. The experimental lateral resolution of PAM is 8.76 μm measured at 600 nm (5.35 μm theoretical). The maximum imaging range of OCT is of 4 mm in air, with a signal attenuation of 3 dB at an axial distance of 1.3 mm measured in air. The supercontinuum source operates at a pulse repetition frequency of 20 kHz and the line camera of the spectrometer is synchronized with the source in order to secure a pulse per integration time (50 μs) and thus per A-scan. Therefore a B-scan frame rate of 20 Hz (500 A-scans) is achieved regardless of the imaging modality employed. The signal generated by the transducer is also synchronized with the excitation pulse; therefore simultaneous OCT-PAM is achieved. However, since PAM detection requires a medium to couple the ultrasound waves, such as water, the absorption at 1310 nm of water limits the OCT capabilities. To demonstrate the maximum capabilities of each imaging modality, for the results presented here, we acquired images with each modality sequentially. No averaging and no post-processing have been applied to images or measurements. To produce the OCT images the complex master slave (MS) method [21], [22] was used. This technique presents the advantage of being tolerant to dispersion left unbalanced in the interferometer and does not require data to be resampled. Moreover, it delivers an absolute value for the axial path in air, allows coherence-limited resolution without any further signal conditioning and can give direct access to any number of *en face* images without requiring to produce the entire OCT volumetric image first. The PAM A-scan is taken as the amplitude envelope of the photoacoustic signal. The OCT images presented here exhibit a signal-to-noise ratio of ∼ 35 dB (system sensitivity: 65 dB), while a signal-to-noise ratio of 20–30 dB is measured on PAM images, depending on the intensity of the signal detected.

To create a phantom with structural information and absorption contrasts that shows the capabilities of our OCT-MPAM system, three different dyes with an absorption spectrum in the visible are mixed with “silicone” (3140 RTV Coating, Dow Corning). The three dyes are Rhodamine B (Flinn Scientific) that has an absorption peak at 542.8 nm, Malachite Green (Sigma–Aldrich) with a peak at 616 nm, and Indocyanine Green (ICG) (Akorn) with a peak at 695 nm and/or 780 nm depending on the concentration. We present images and hemoglobin oxygen saturation measurements from an *in vitro* mouse ear to illustrate the system's potential for biological tissue. Animal tissue was obtained from C57BL/6J mice purchased from Charles River Laboratories (Margate, UK). Mice were humanely culled in accordance with the UK Home Office guidelines and following approval by the University of Kent's animal welfare ethics committee prior to ear tissue removal.

## 3 Results and discussion

We present spectroscopic photoacoustic measurements for different samples offering different absorption spectra in the visible as well as OCT-MPAM for different *in vitro* samples: synthetic phantoms and a mouse ear.

### 3.1 Spectroscopic photoacoustic

**Fig. 4:**
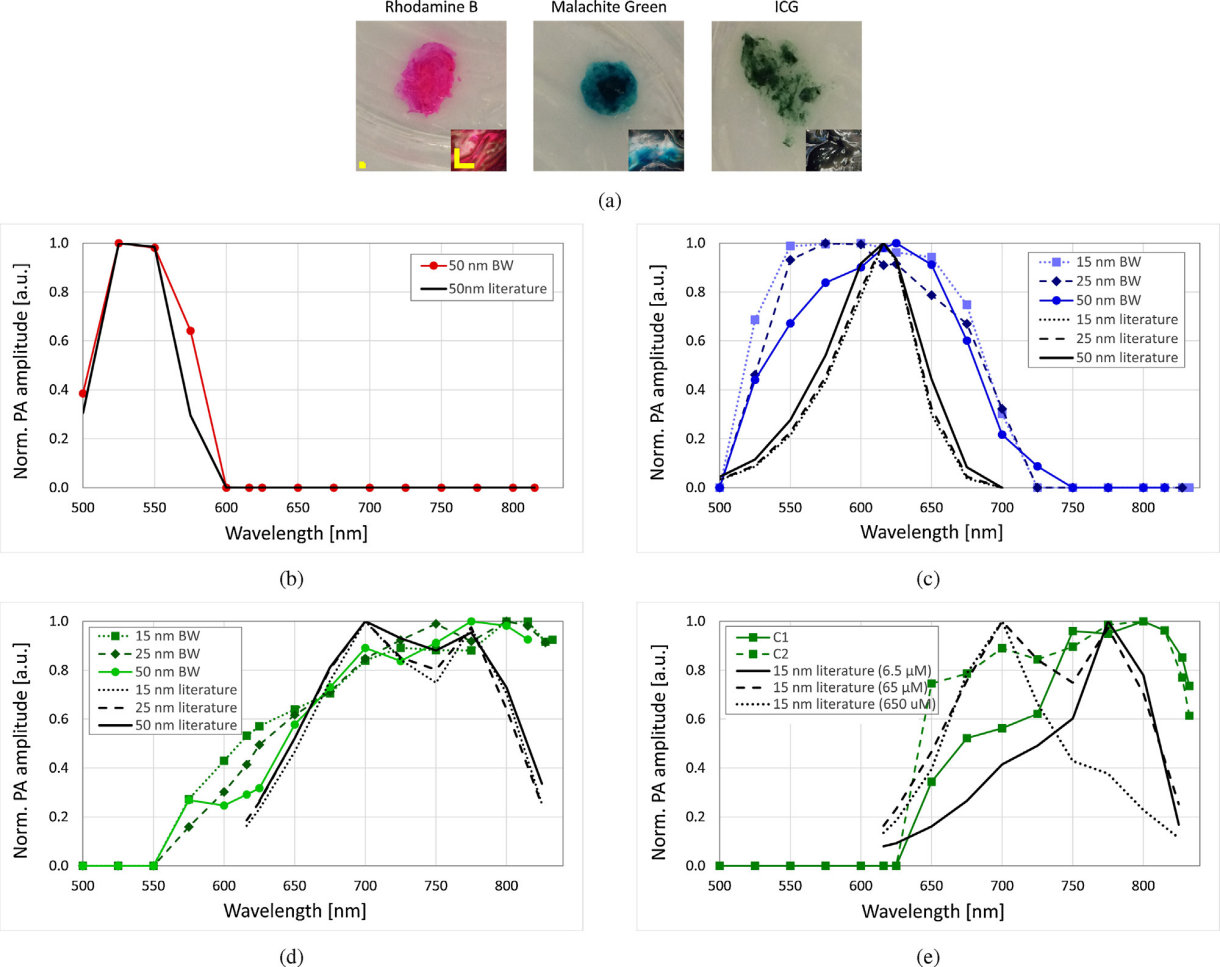
(a) Photo of the three different samples, with microscopic image by reflection, scale bars 1 mm. Absorption spectrum adapted from the literature and inferred via photoacoustic measurements for different bandwidths (15 nm, 25 nm and 50 nm) of (b) Rhodamine B, (c) Malachite Green, (d) Indocyanine Green (ICG) (with literature corresponding to a concentration of 65 μM) and (e) ICG for different concentrations with 15 nm bandwidth.

To demonstrate the spectroscopic capabilities of our visible photoacoustic system, measurements were made at a single position of a silicone-dye sample, as shown in Fig. 4(a). An oscilloscope was used to register the amplitude of the acoustic pulse for each wavelength. For a given absorption of a sample, the response of the transducer scales linearly with the pulse energy on the sample for energy values larger than 1 nJ and up to at least 100 nJ. Therefore we normalized our measurements over the energy on the sample since this parameter varies with the wavelength. In addition, for each dye, the absorption curve has been normalized to its maximum value, therefore we are not presenting any quantitative measurements in terms of concentration here, but simply the absorption spectra. We have also studied the effect of the bandwidth (linked to the energy) on the accuracy of spectroscopic measurements, employing 15 nm, 25 nm and 50 nm bandwidths. This study was done only with Malachite Green and ICG. In Figs. 4(b)–(d) the photoacoustic measurements carried out with different bandwidths of Rhodamine B, Malachite Green and ICG respectively, are compared to the absorption curves adapted from literature in ethanol, water and water, respectively [23], [24], [25]. The “literature” curves in Fig. 4 are constructed using data retrieved from references [23], [24], [25]. These have been integrated over a specific bandwidth (15 nm, 25 nm or 50 nm) in steps of 25 nm in order to ensure a fair comparison between our photoacoustic measurements and the literature for each of the absorbers. The absorption spectrum depends on the media the dye has been diluted into; in our case no literature reference is available for silicone. The spectra of Malachite Green and ICG measured are broader and flatter than found in the literature. We conjecture that this is due to the influence of silicone on the dyes absorption and/or due to the fact that in our phantoms, the dyes aggregate in the silicone, as observed in Fig. 4(a). Aggregates have different properties than uniformly dissolved materials [26]. Figs. 4(b)–(d) show that the spectroscopic measurements can resolve the absorption spectra of different absorbers within the 500–840 nm range. Our system is capable of distinguishing between different absorbers within this range. The spectral bandwidth of the excitation light source has no significant influence on the measurements. By performing the photoacoustic measurements three times at the same position on the sample, an uncertainty of ∼1–20% has been estimated.

Fig. 4(e) shows the spectroscopic photoacoustic measurements, performed every 25 nm with a bandwidth of 15 nm, at two different positions of the ICG sample, which correspond to two different unknown concentrations (C1 and C2). The ICG absorption spectrum depends on the concentration. In Fig. 4(e) three different absorption curves of ICG corresponding to three ICG concentrations in water from literature [24], [25] are represented (integrated over 15 nm-wide bands). The sample with an unknown concentration C1 (respectively C2) presents an absorption spectrum similar to the spectrum corresponding to a concentration of 6.5 μM (respectively 65 μM). This shows that we can differentiate between two different ICG concentrations within the same phantom.

### 3.2 *In vitro* OCT-MPAM

**Fig. 5:**
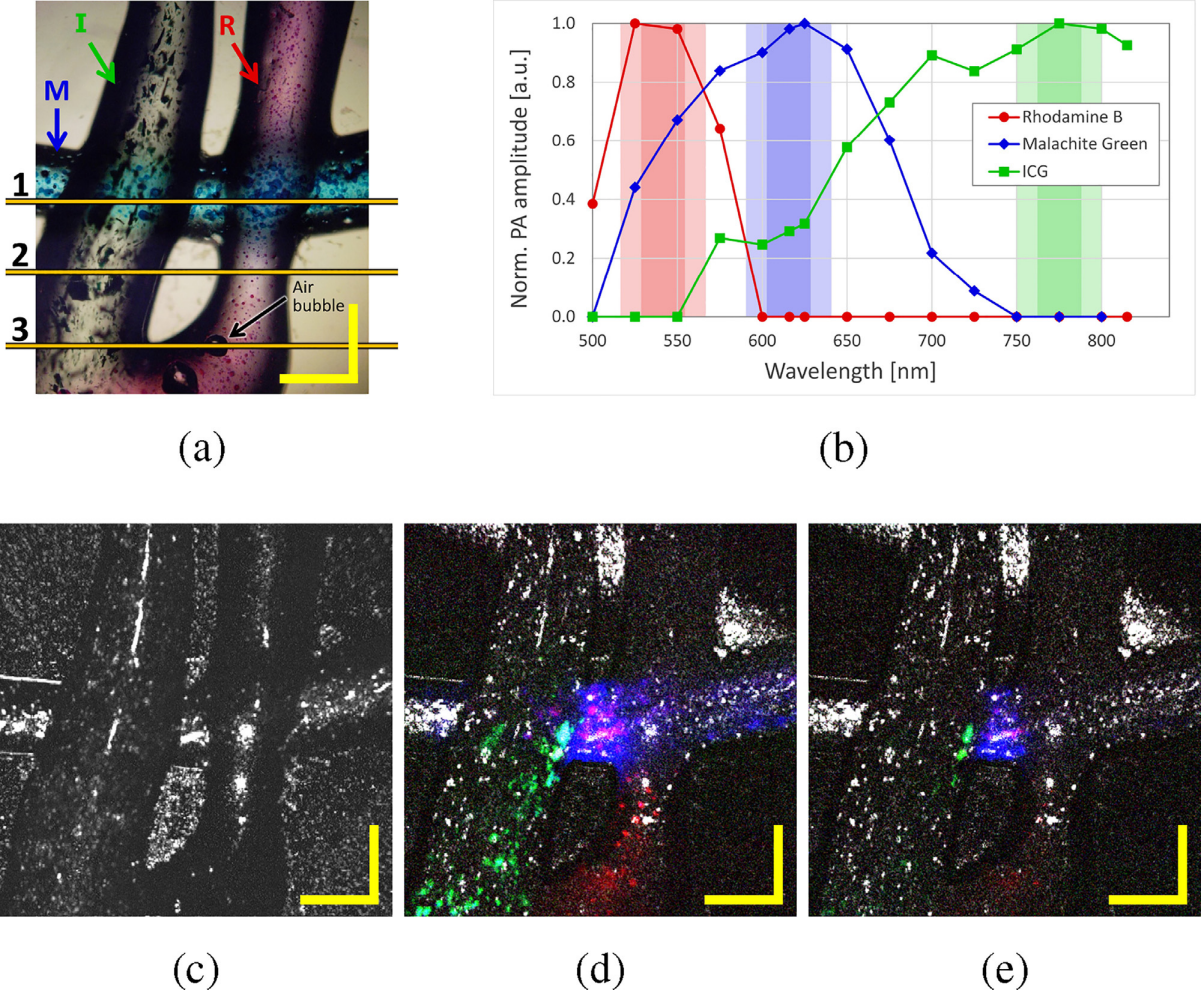
(a) Sample under the microscope (×50) with Rhodamine B (R), Malachite Green (M) and ICG (I), (b) absorption spectra of the three dyes replicated from Figs. 4(a)–(c) in relation to the spectral bands to be used for MPAM excitation, of 25 nm and 50 nm width (shown by coloured bars), (c) OCT summed voxel projection along axial direction for 3 mm in air, (d) superposition of *en face* OCT at position marked in Fig. 6(b) (grey) and PAM maximum amplitude projections at 542 nm, 616 nm and 800 nm with 50 nm bandwidth (red, blue and green, respectively) and (e) superposition of *en-face* OCT and PAM maximum amplitude projections with 25 nm bandwidth. Scale bars 500 μm.

*In vitro* imaging capabilities of the bimodal OCT-MPAM system were tested on a silicone-based sample consisting of the three dyes mentioned above. Fig. 5(a) shows a microscope view of the sample, where the Malachite Green is situated below the Rhodamine B and the ICG. It should be noticed that the concentration of dyes within the sample is not uniform and aggregates formed. This is important to retain for the later interpretation of PAM signals from different parts of the sample, as labelled by 1, 2, 3. Fig. 5(b) shows the absorption spectra measured in Section 3.1 corresponding to the three different dyes. PAM was performed on the silicone-based sample at three different wavelengths: 542 nm, 616 nm, and 800 nm using a bandwidth of 50 nm in each case. The influence of the bandwidth has also been studied by doing the same measurements with a reduced bandwidth of 25 nm. The total area scanned by both modalities is 2.5 mm × 2.5 mm and consists of 500 × 500 A-scans. The OCT images correspond to the sample without water, while for PAM water has been introduced to couple the ultrasound waves to the transducer. No post-processing has been applied to the OCT images. For PAM, each A-scan is the envelope of the acoustic signal detected. The detection configuration used for PAM induces a curvature in the PAM B-scans. The travelling time to the transducer from a point in the center of the sample is shorter than the travelling time from an off-axis point. A linear deformation is observed in the other lateral direction since the transducer is situated close to the edge of the sample. The travelling time of the acoustic wave from a point on a sample situated at the opposite edge is longer than that from a point close to the edge where the transducer is positioned. We corrected for these deformations and adjusted the contrast of the images to offer a better representation to the reader.

**Fig. 6:**
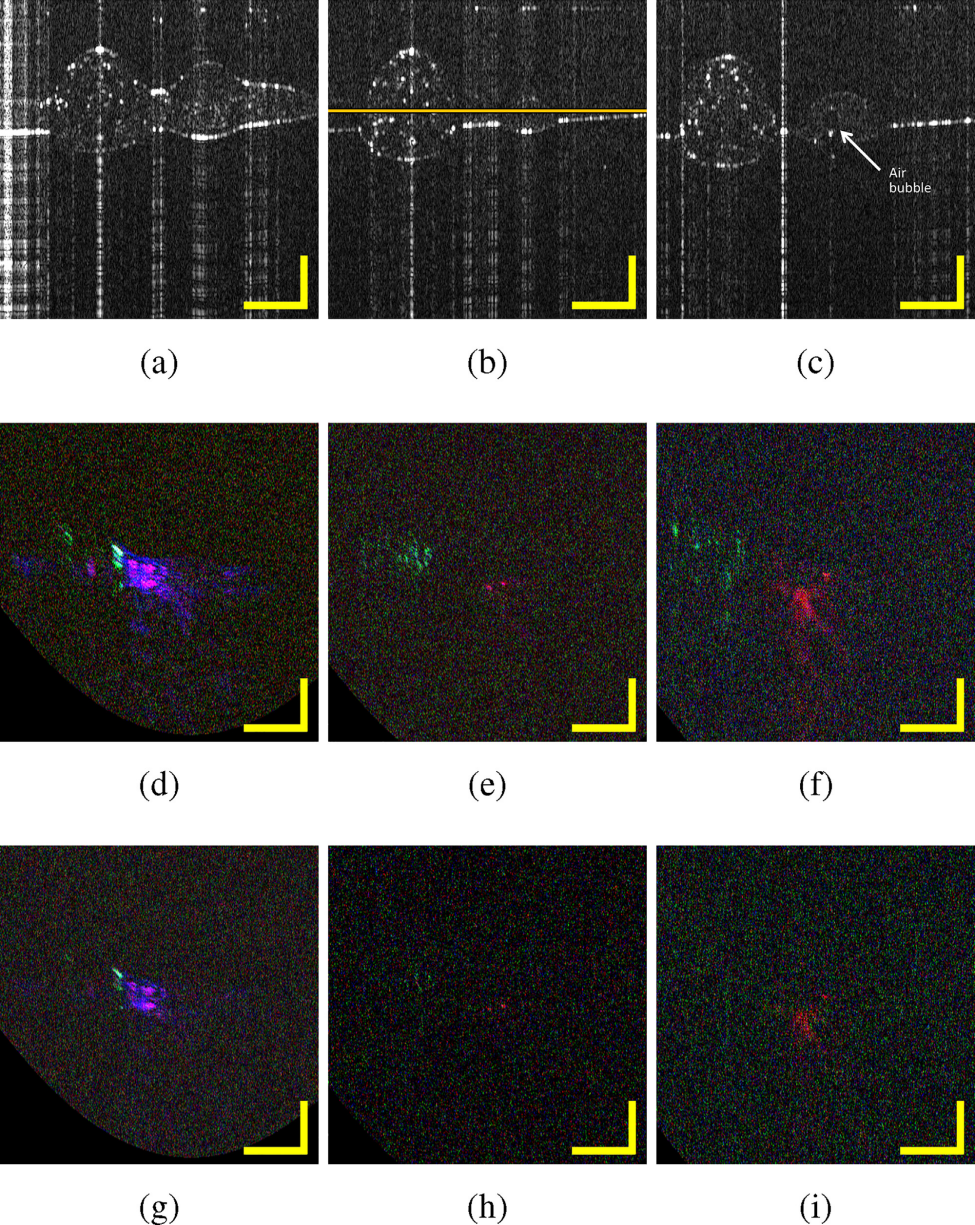
(a)–(c) OCT B-scans corresponding to respectively the positions 1, 2 and 3 marked in Fig. 5(a), (d)–(f) superposition of PAM B-scans at 542 nm, 616 nm and 800 nm with 50 nm bandwidth (red, blue and green, respectively) at positions 1, 2 and 3 respectively, (g)–(i) with 25 nm bandwidth at position 1, 2 and 3 respectively. Scale bars 500 μm.

Fig. 5(c) is the summed voxel projection of the OCT for all depths (3 mm in air). Fig. 5(d) represents the superposition of the *en face* OCT image corresponding to a single depth (at 1.1 mm measured in air) as indicated in Fig. 6(b), with the PAM maximum amplitude projections taken at 542 nm, 616 nm and 800 nm for bandwidths of 50 nm. The OCT is represented in grey scale while the red corresponds to PAM with the band centered at 542 nm, blue at 616 nm and green at 800 nm. Purple results from superposition of red and blue colours corresponding to the overlay of PAM signals at 542 nm and at 616 nm, cyan results from superposition of blue and green corresponding to an overlay of PAM signals at 616 nm and 800 nm. Fig. 5(e) corresponds to the same PAM images employing a bandwidth of 25 nm, to narrow the spectral excitation.

B-scans images in columns 1, 2 and 3 of Fig. 6 are collected at different lateral positions indicated respectively by the lines 1, 2 and 3 over Fig. 5(a). Figs. 6(a)–(c) represent the OCT B-scans; the bright vertical lines are due to camera saturation induced by strong direct reflection of the light from the sample. Figs. 6(d)–(f) show the PAM B-scans obtained for a bandwidth of 50 nm, and Figs. 6(g)–(i) for a bandwidth of 25 nm. The B-scans corresponding to each band have been overlaid with the same colour code used Figs. 5 (d) and (e): red for 542 nm, blue for 616 nm and green for 800 nm.

OCT is a suitable tool to image the sample structure, however its limitations can be seen in Figs. 5(c)–(e) and 6 (a)–(c), where the different absorbers cannot be differentiated because of their similar structure and index of refraction. MPAM provides additional functionality to the system by allowing this differentiation between absorbers.

In Figs. 6(d) and (g) the signals originating from Malachite Green and ICG are strong. Rhodamine B is strongly absorbing in the band centered at 542 nm but is not detected here. Therefore, we conclude that despite the large absorption of Rhodamine B in this band, its concentration at position 1 is too low to be detectable by the system. In Figs. 6(e), (f), (h) and (i) we can observe as expected the Rhodamine B, and additionally some artefacts in Figs. 6(f) and (i) corresponding to an air bubble in the sample that can be seen in Fig. 5(a) and in the OCT B-scan in Fig. 6(c). Because the excitation band centered at 616 nm overlaps with the wing of the absorption spectrum of the ICG absorption spectrum extending from its central peak at 800 nm, areas of high ICG concentration contribute to absorption at 616 nm, and generate a PAM signal. Superposition of green (PAM signal due to absorption at 800 nm) and of blue (PAM signal due to absorption at 616 nm) leads to the cyan coloured areas displayed in Fig. 5(d). The bandwidth centered at 800 nm does not present any overlapping with the other two dyes absorption spectra, as observed in Fig. 5(b). Therefore, as expected, in Fig. 5(d) and (e) and 6, only ICG contributes to the PAM signal when excited with the band centered at 800 nm. It can be observed in Fig. 5(a) that the dyes form aggregates within the silicone, that leads to variation of concentration and consequently of the OCT and PAM signal across the sample. This is observed in the PAM and OCT images in Figs. 5 and 6 .

This combined OCT-MPAM system offers complementary information and gives functional information from the sample, and allows distinction between different absorbers within the 500–840 nm range. To distinguish more accurately between the different absorbers, additional spectral bands can be used as well as reducing the bandwidth of the excitation beam. However, reducing the bandwidth leads also to a lower energy on sample and therefore the detected signal becomes weaker and the effective imaging area is further limited. However, in Figs. 5(e) and 6 (g)–(i), the overlapping of the 25 nm bands due to adjacent absorbers is less observed than with the 50 nm bands (Fig. 5(d)). Reducing the bandwidth gives more accurate spectral characterization of the absorber at the cost of a narrower field of view since the energy on sample is reduced. The use of more wavelengths would therefore be more suitable to improve the accuracy of the measurements.

### 3.3 *In vitro* OCT-MPAM of a mouse ear

**Fig. 7:**
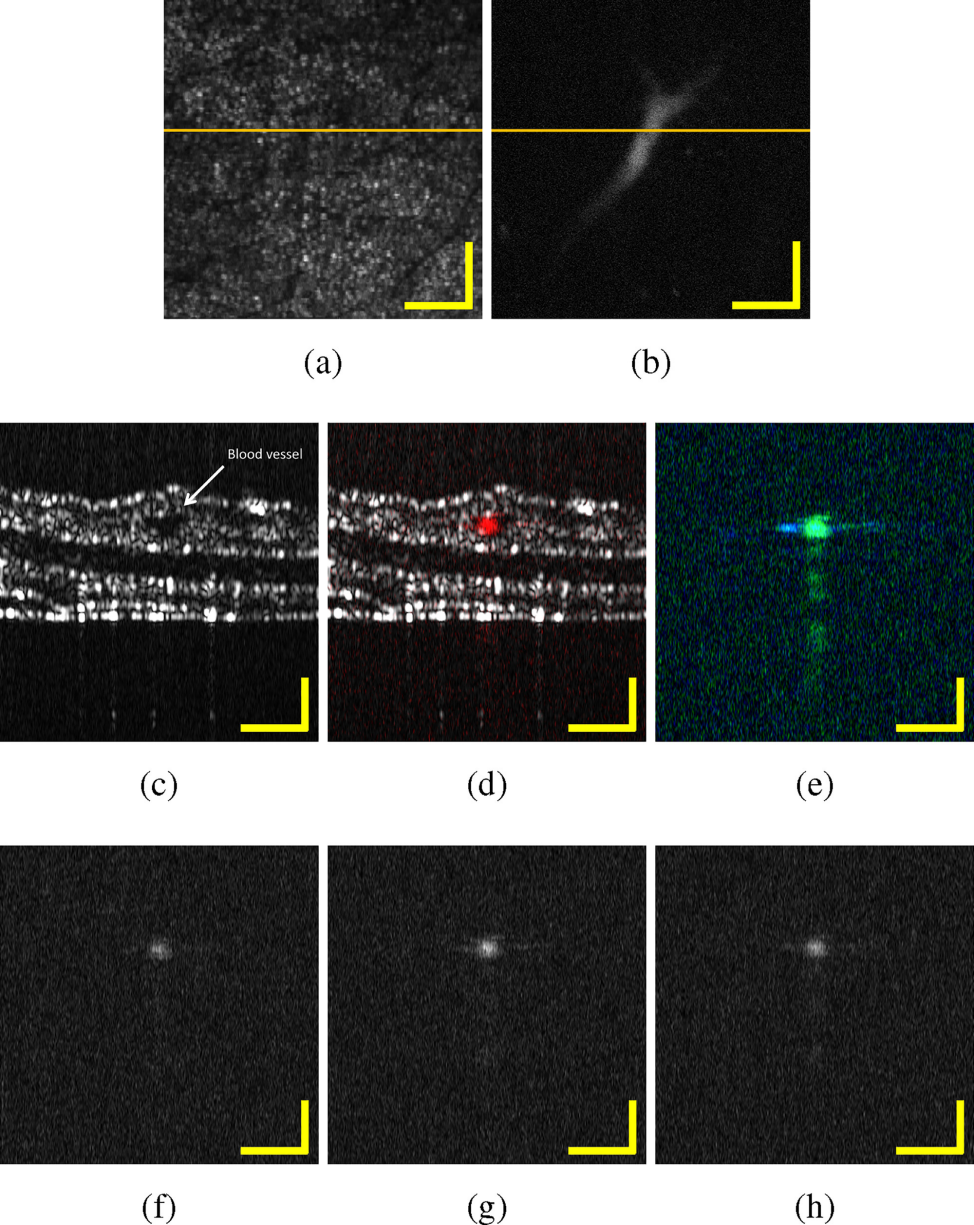
*In vitro* mouse ear (a) OCT summed voxel projection along axial direction for 2 mm in air, (b) PAM maximum amplitude projection at 550 nm with 50 nm bandwidth, (c) OCT B-scan, (d) superposition of OCT B-scan (grey) and PAM B-scan at 550 nm with 50 nm bandwidth (red), (e) superposition of PAM B-scans for bandwidths covering 500–600 nm and 600–840 nm (green and blue, respectively), (f)–(h) PAM B-scans for a bandwidth of 50 nm with a central wavelength of respectively 530 nm, 550 nm and 580 nm. All B-scans are taken at the position indicated on (a) and (b). Scale bars 250 μm.

**Fig. 8:**
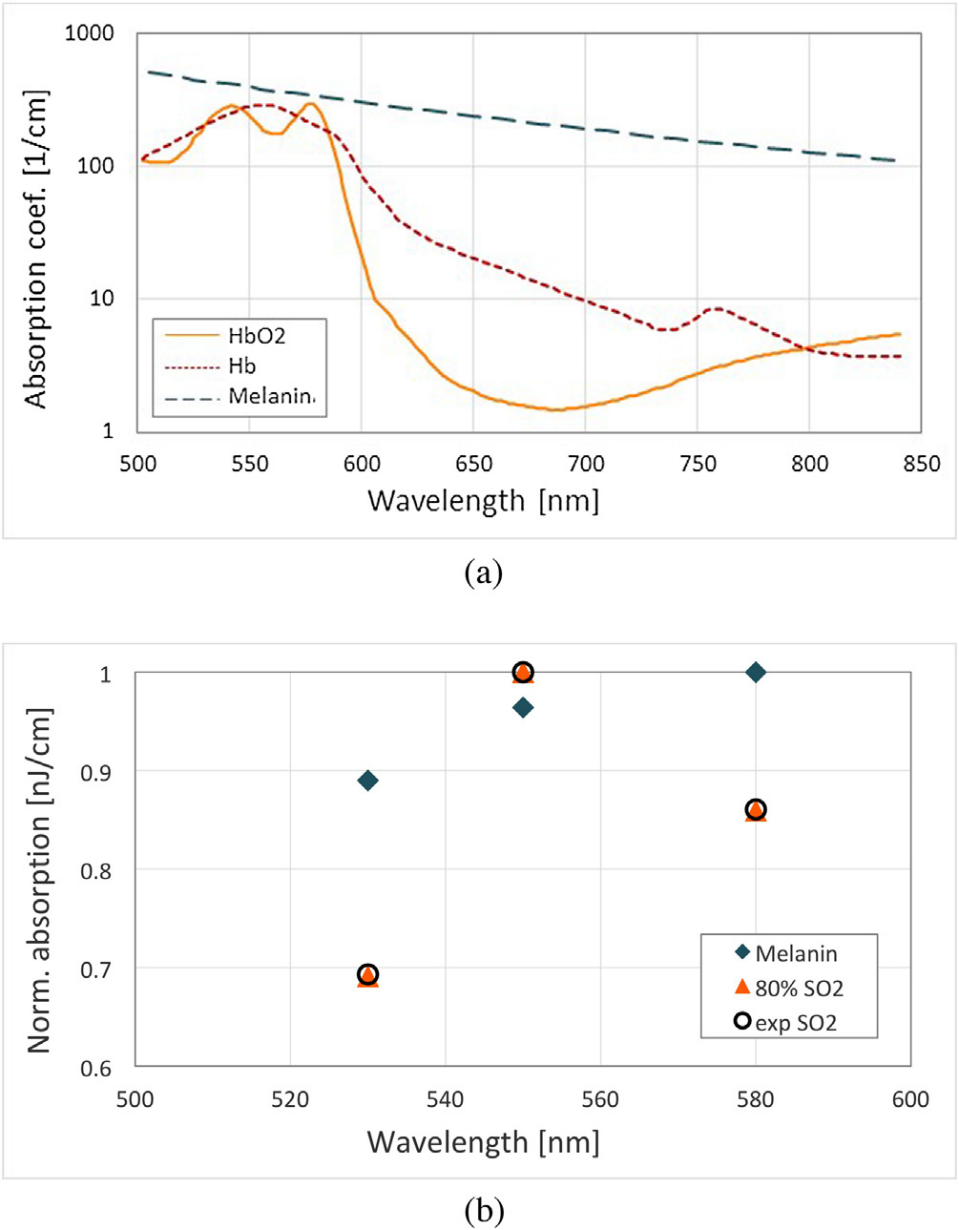
(a) Absorption coefficients of oxygenated and deoxygenated hemoglobin [27] and melanin [28] and (b) theoretical and experimental PAM normalized amplitude of these absorbers in the region of interest.

Images of blood vessels were taken from an excised mouse ear. Given the nature of the *in vitro* preparation we were only able to successfully image lager capillaries due to the likely collapse of the smaller vascular tubular networks which are typically present in the ear. Fig. 7 shows the images generated by OCT and MPAM. PAM has been registered for multiple bands: first with bands extending from 500 nm to 600 nm, then from 600 nm to 840 nm, then for different central wavelengths: 530 nm, 550 nm and 580 nm, in each case with a bandwidth of 50 nm. Fig. 7(a) represents the OCT summed voxel projection, with the pigmentation of melanin as well as some glands being observed. A large blood vessel is imaged by PAM in Fig. 7(b) using the PAM maximum amplitude projection at 550 nm with a bandwidth of 50 nm. The OCT B-scan, shown in Fig. 7(c), demonstrates the different layers of the mouse ear and the large blood vessel in the middle. The presence of the blood vessel is confirmed in Fig. 7(d) that represents the overlay of the OCT B-scan and the PAM B-scan taken from Fig. 7(a) and (b), respectively, at the position indicated by the line. The absorption of the skin (melanin) can also be observed with PAM depending on the wavelength range used. Fig. 8(a) shows the absorption coefficients of oxygenated and deoxygenated hemoglobin [27] (*HbO*_2_ and *Hb*) and melanin [28] over our spectral range of interest: 500–840 nm. Hemoglobin and melanin, the two main constituents of our *in vitro* sample, absorb strongly in our region of interest. This is observed in Fig. 7(e) which represents the coloured overlay of PAM B-scans taken for the 500-600 nm band (green) and for the 600–840 nm band (blue). Cyan results from a superposition of green and blue corresponding to an absorption in both bands (i.e. melanin, see Fig. 8(a)). For wavelengths longer than 600 nm, melanin is the strongest absorber (blue in Fig. 7(e)) while in the range extending from 500 nm to 600 nm, both melanin and hemoglobin manifest absorption (cyan and green, respectively). Furthermore, we have studied the main region of interest for blood (500–600 nm). Figs. 7(f)–(h) represent the PAM B-scans for a central wavelength of 530 nm, 550 nm and 580 nm respectively. In Fig. 8(b), the experimental data (black circles) are presented: for each wavelength, the maximum amplitude of the B-scan is taken and normalized over the maximum obtained; corresponding to the value at 550 nm. The theoretical amplitudes of the expected photoacoustic signals for melanin and for hemoglobin are superposed to the experimental values in Fig. 8(b). We took into consideration the amount of energy incident on the sample as a function of the central wavelength to normalize our absorption coefficients. In addition, for each absorber, we normalized these values over their maxima. In Figs. 7(f)–(h) we should expect similar signal amplitude for the melanin layer (horizontal line) at each of the three wavelengths. The data set corresponding to a theoretical oxygen saturation of 80% has been found to be the best match to the experimental values. The blood vessel imaged here presents an oxygen saturation of approximately 80 ± 5%.

We have shown the concept of MPAM on *in vitro* biological samples combined with OCT. Hemoglobin and melanin are clearly distinguishable with PAM by using different spectral bands of the supercontinuum excitation beam.

## 4 Conclusion

Bimodal imaging OCT-MPAM is demonstrated using a single compact supercontinuum source. Operation in a wide range extending from 500 nm to 840 nm is proven here for multispectral measurements. The capabilities of each modality for real-time imaging were demonstrated on several samples, *in vitro*. OCT offers structural information at 1300 nm, however OCT is often not sufficient to differentiate absorbers. As a complement MPAM using a supercontinuum source is a versatile tool for measuring absorption spectra, oxygen saturation and differentiate absorbers within the 500–840 nm range. Increasing the number of wavelengths used for PAM will improve the accuracy of the results. These studies can be extended to *in vivo* biomedical samples.

## Conflict of interest

The authors declare no conflicts of interest.
